# EFFECTS OF AN AGE SUIT SIMULATION ON NURSING STUDENTS’ PERSPECTIVES ON PROVIDING CARE TO OLDER PERSONS

**DOI:** 10.1093/geroni/igad104.2422

**Published:** 2023-12-21

**Authors:** Jenny Hallgren, Björn Bouwmeester Stjernetun, Catharina Gillsjö (Gillsjo)

**Affiliations:** University of Skövde, Skövde, Vastra Gotaland, Sweden; University of Skövde, Skövde, Vastra Gotaland, Sweden; University of Skövde, Skövde, Vastra Gotaland, Sweden

## Abstract

**Background:**

Nursing students are important future health care providers to the growing number of older persons in society. However, two barriers are their common ageist attitudes and lack of interest in geriatrics. This is a concern in light of the global demand for more nurses. Method: This study investigated the effects of ageing simulation with an age suit as a part of experiential learning in a nursing programme among 471 nursing students. The simulation allowed the students to experience specific and common health problems from the patient’s point of view in a controlled environment and a relevant context of care provision for future nurses: a home with welfare technology and other aids. The learning process involves a continuous cycle of doing and reflecting resulting in knowledge that also becomes “ingrained” in the body of the learner. Data on the Perspectives on Caring for Older Patients – Short Form (PCOP-SF) scale were collected using a quasi-experimental pretest–posttest design with a control group.

**Results:**

The results showed that the intervention had a positive effect on various aspects of nursing students’ perspectives on caring for older persons. Work experience was associated with more positive attitudes. The control group was more negative towards geriatrics as a career choice than the intervention group.

**Conclusion:**

Age suit simulation can be an innovative intervention in nurse education as it raises awareness and understanding of aging and the health challenges of older persons, which are important in combatting ageism among future nurses.

